# Professor Johannes Vester, President of the Academy for Multidisciplinary Traumatology: Adapted Interview from the 20^th^ AMN Congress (May 12-13), Krakow, Poland

**DOI:** 10.25122/jml-2023-1023

**Published:** 2023-06

**Authors:** Alexandra Gherman, Andreea Strilciuc

**Affiliations:** 1 RoNeuro Institute for Neurological Research and Diagnostic, Cluj-Napoca, Romania

**Interviewer: Alexandra GHERMAN**

**Interviewee: Professor Johannes VESTER°**

**°** Invited Associate Professor, Department of Neurosciences, Iuliu Hatieganu University of Medicine and Pharmacy, Cluj-Napoca, Romania

Head Biometry and Clinical Research

idv - Data Analysis and Study Planning, Germany

**A.G**.: Dear Professor Johannes Vester, as President of the Academy for Multidisciplinary Neurotraumatology (AMN), could you please share with us your thoughts about this organization upon reaching the important milestone of its 20^th^ Congress?

**J.V**.: Thank you very much for that question! You know, after that long pause due to Covid19 -the last Congress was in 2020 in Egypt, we all realized here again the importance of face-to-face communication to really move things forward and to fully use available synergies. That’s so important for the benefit of future patients. This interdisciplinary sharing of scientific perspectives, in an open, respectful framework, as we do it here, is of crucial importance for a better understanding of the really complex nature of neurotrauma, especially of traumatic brain injury, and also for developing successful new treatment concepts, by putting all these aspects together. Because neurotrauma is multidisciplinary, it is multidimensional from its very nature. And AMN, as the Academy for Multidisciplinary Neurotraumatology, is dedicated to opening the mindsets to this, to communicate the breadth of perspectives necessary to capture that whole picture of neurotrauma and recovery. And maybe as a message for our colleagues here present at the 20^th^ Congress, I would like to say that it is extremely important to develop new perspectives for the future on the highest scientific levels, sharing the experience of all the experts with the audience, learning from the past, and communicating to clinical practice, and also, very important, closing the feedback cycle by listening very carefully to the local practitioners. So, this is really a coming together of all this information from the top scientific level to bottom-down local conditions – it has to be a living feedback cycle to really move things forward. That is our aim!

**A.G**.: In connection to your answer, the Academy is currently working on the set-up for a new guideline, in partnership with several affiliated societies. What will be the priority topics that these guidelines will address and what added value will they bring to the Academy’s institutional network?

**J.V**.: Thank you very much for this question! Well, as I said, due to the complexity and vast heterogeneity of neurotrauma, especially in traumatic brain injury, I would say that many clinicians are left alone in terms of modern guidelines in this field based on evidence-based pillars. So, while there exist a lot of neurosurgical management guidelines, there exists, to our knowledge, no treatment guideline yet incorporating the complex multidimensional nature of TBI in the early recovery phase initiating treatment options, e.g. in the first 24 hours. And, as stated in the most recent CENTER-TBI elaboration on the sensitivity of outcome instruments after TBI, published this year, it is recommended that multidimensional outcome assessments are performed for a comprehensive representation of a patient’s clinical picture. This is a very important aspect. It requires all of us to make a big step forward from a single outcome assessment approach, which can only look from a narrow perspective at the patient - that was the methodology of the past, to address the multiplicity of important domains such as physical function, cognitive function, emotional and mental health, global functional outcome and so on. So, to close this gap, we will focus in this guideline on these most relevant domains of interest for all patients undergoing early recovery after traumatic brain injury. And, of course, the aim is to help clinical decision-making by any healthcare professional involved in TBI neurorehabilitation.

**A.G**.: Professor Vester, thank you for this most comprehensive answer. On a scale from 1 to 5, where would you place the implementation of GRACE Principles for High-Quality Observational Studies of Comparative Effectiveness in TBI research?

**J.V**.: Nice question, easy answer! Score 5. Why? As you know, in observational trials in terms of the GRADE framework where the evidence is rated if it comes to treatment recommendations, the observational trial ‘enters the door’ with a ‘low evidence rating’, that is the starting point. Now, in practice, observational trials are not so good, [they are] mostly controlled, there is a lot of lost follow-ups to patients and so, they are further down-graded from ‘low’ to ‘very low’ or ‘no evidence’. What does that mean? We invest ideas, resources, energy, dedication, passion, and financial grants to observational trials which, in the end, have no chance to be of any value to the community when it comes to rating the quality of evidence because they are rated with ‘very low’ or ‘no evidence’ at all. So, when it comes to guideline production, they have no value and they are left with the final statement ‘no evidence’. That is why [the] score 5 for implementation of the High-Quality principles to observational studies.

**A.G**.: What do you believe are the main elements of the modern multidimensional approach that any TBI researcher should be guided by?

**J.V**.: Yes, this is an important question. The outcome of TBI is by its very nature multidimensional. Human beings are multidimensional. There is not only a physical function to be restored, but there is also emotional and mental health, and there is social reintegration - a variety of aspects important for the quality of life for the individual human being affected by a traumatic brain injury. And, unfortunately, there is still a widespread treatment nihilism about cognitive, behavioural and depressive disorders in acute care. This has to be changed. We have to change that if we want to effectively bring the patient back to what he has been before the trauma. All these aspects play a major role for the individual [human] being and we have to open up our focus, all of us, to the breadth of these long-term impairments that are so important for the patient and also his relatives and family, and beloved ones.

**A.G**.: Thank you very much! Last but not least Professor Vester, what are the main future developments the Academy for Multidisciplinary Neurotraumatology is focused on, both short- and long-term?

**J.V**.: I would say, first of all, and basic focus of the Academy is still to further open the doors between the academic disciplines, to bridge, to take away the walls which inhibit that we really share knowledge and come to a bigger and more successful picture and potential to help the patients. So, this intense multidisciplinary exchange of knowledge and different perspectives with the aim to establish effective pathways for the treatment of neurotrauma patients. That is the basis of this multidisciplinary Academy. And a second focus crystallized most recently: to promote modern research approaches, to help them to come to life, interventional or non-interventional, by providing in-depth methodological support and by offering the umbrella of AMN to endorse promising future-orientated clinical research. In this way, AMN is seeding projects from which new therapeutic options are expected and which address the breadth of potential deficits and recovery following neurotrauma. And maybe a third focus to mention – as we are all here, is the enlargement of the educational and teaching activities that are also very important, to keep a living feedback cycle. The Neurotrauma Simulation Center was already successfully implemented last year in Vienna, also this year, with participants from 6 low-income countries, with a variety of disciplines – neurosurgeons, neurologists, psychiatrists, psychotherapists, and so on, all exchanging their knowledge and their experience. This teaching domain will be further strengthened and extended in the future so that AMN might become a kind of key transmitter of interdisciplinary knowledge in neurotrauma. All in all, there are a lot of exciting developments and a lot of work still to do. We encourage young clinicians and researchers to join our organization and become part of this exciting AMN academic network. Thank you very much!

**A.G**.: Professor Vester, thank you very much for this interview and I wish you the best of luck in all your endeavours!

**J.V**.: Thank you so much, you are welcome!

